# Malaria determining risk factors at the household level in two rural villages of mainland Equatorial Guinea

**DOI:** 10.1186/s12936-018-2354-x

**Published:** 2018-05-18

**Authors:** Mónica Guerra, Bruno de Sousa, Nicolas Ndong-Mabale, Pedro Berzosa, Ana Paula Arez

**Affiliations:** 1 Global Health and Tropical Medicine, GHTM, Instituto de Higiene e Medicina Tropical, IHMT, Universidade Nova de Lisboa, UNL, Rua da Junqueira 100, 1349-008 Lisbon, Portugal; 2 Faculdade de Psicologia e de Ciências da Educação, CINEICC, Universidade de Coimbra, Coimbra, Portugal; 3 Centro de Referencia para el Control de Endemias, Instituto de Salud Carlos III, Bata, Equatorial Guinea; 4 Centro Nacional de Medicina Tropical, Instituto de Salud Carlos III, Madrid, Spain

**Keywords:** Malaria infection, *Plasmodium* spp., Risk factors, Socioeconomic status, Equatorial Guinea

## Abstract

**Background:**

After the introduction of an artemisinin-based combination therapy, the reduction of prevalence of malaria infections has shown a remarkable progress during the last decade. However due to the lack of a consistent malaria control programme and socioeconomic inequalities, *Plasmodium* infection is still one of the major cause of disease in Equatorial Guinea, namely in the rural communities. This study explored the associated risk factors of malaria transmission at the microeconomic level (households) in two rural villages of mainland Equatorial Guinea.

**Methods:**

This survey involved 232 individuals living in 69 households located in two rural villages, Ngonamanga and Miyobo, of coastal and interior of Equatorial Guinea, respectively. Malaria prevalence was measured by PCR and parasitaemia level by optical microscopy; household socioeconomic status (SES) was measured based on house characteristics using a 2-step cluster analysis. Logistic regression analysis was performed to investigate the relationship of a diverse set of independent variables on being diagnosed with malaria and on showing high levels of parasitaemia.

**Results:**

The prevalence of *Plasmodium* spp. infection was 69%, with 80% of households having at least one parasitaemic member. The majority of houses have eaves (80%), walls of clay/wood (90%) and zinc roof (99%) and only 10% of them have basic sanitation facilities. The studied areas showed reduced rates of indoor residual spraying coverage (9%), and long-lasting insecticide-treated net ownership (35%), with none of these preventive tools showing any significant effects on malaria risk in these areas. Neither the risk of malaria infection (PCR positive result) or the development of high parasitaemia did show association with SES.

**Conclusions:**

This study has contributed to reinforce the importance of living conditions associated to a high risk of malaria infection and vulnerability to develop high parasitaemia. This study also contributes to future malaria control interventions to be implemented in mainland Equatorial Guinea or in other countries with similar environmental conditions.

**Electronic supplementary material:**

The online version of this article (10.1186/s12936-018-2354-x) contains supplementary material, which is available to authorized users.

## Background

A significant decline in global malaria burden have been reported since 2000, mainly attributed to scale-up of interventions including indoor residual spraying (IRS), high coverage of long-lasting insecticide-treated net (LLIN), improvement of diagnostic testing and use of artemisinin-based combination therapy (ACT) [1]. However, globally, this parasitic disease persists as one of the deadliest in the world with 445,000 deaths estimated in 2016 and 216 million new cases, particularly in Africa, where 91% of the fatal cases occur [1].

Malaria burden depends on intrinsic (human, parasite, mosquito) and extrinsic (environmental, social, behavioural, political, economic conditions and disease-control efforts) factors, being the latter the most important and relevant one of the determinants for malaria control and prevention [2]. A recent published study, carried out in approximately the same region reported the influence of environmental factors, namely altitude and distance to rivers and forests, as risk factors of malaria infections, mainly associated to higher exposure to mosquito vector [3].

Associated risk factors of malaria at the microeconomic level were analysed in the context of a study which integrated analysis of malaria prevalence, molecular characterization of *Plasmodium* populations (anti-malarial resistance-associated mutations) and characterization of malaria risk determinants in two rural villages, Ngonamanga and Miyobo, of mainland Equatorial Guinea. Previous analysis hinted differences between the two villages, regarding frequency of anti-malarial resistance associated mutations and *Plasmodium falciparum* diversity associated to different socioeconomic characteristics, populations migration and different accessibilities to anti-malarial treatment [4]. In the present report, the association of socioeconomic status of families/households with local malaria burden was evaluated.

**Fig. 1:**
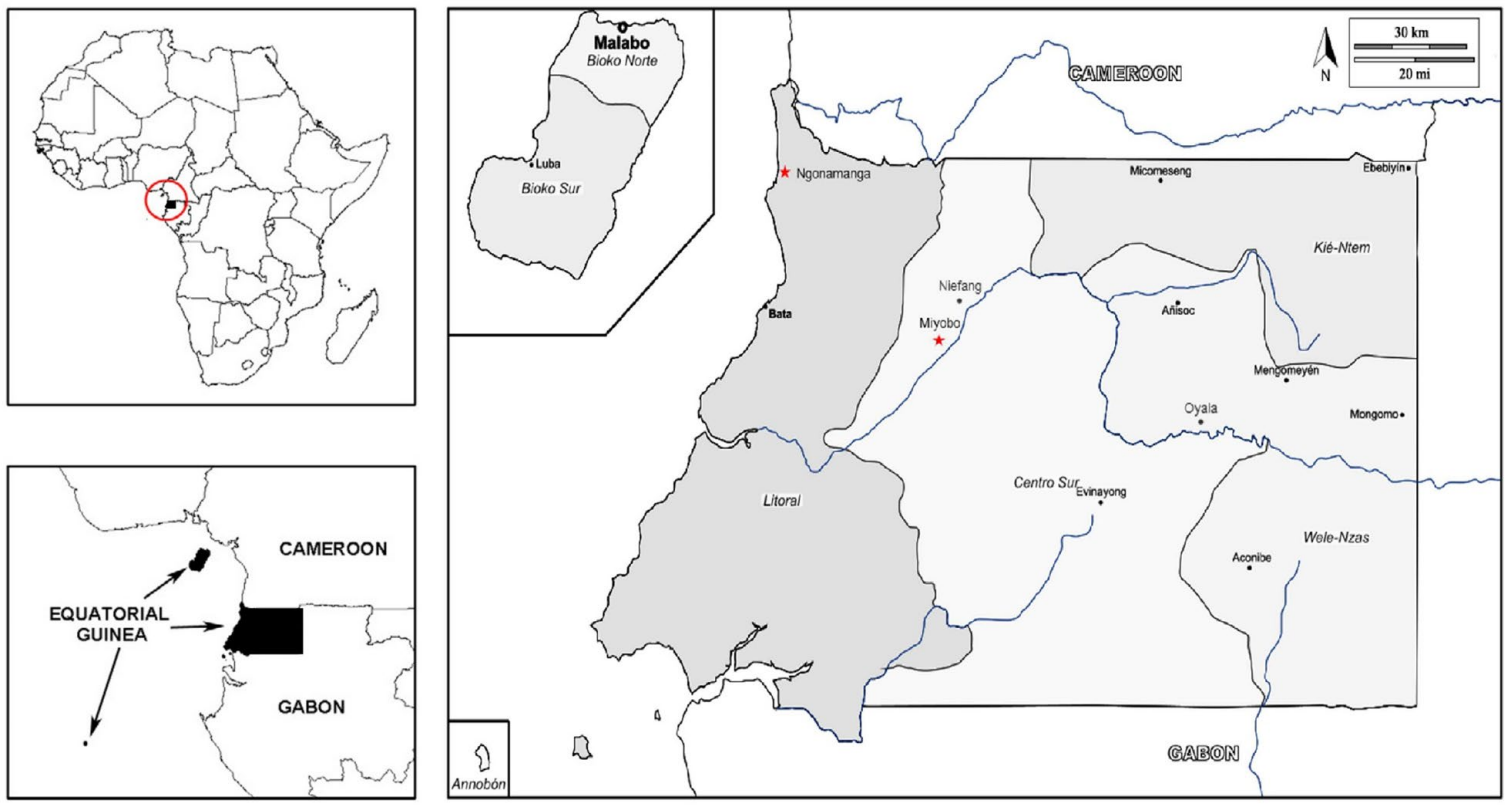
Map of Equatorial Guinea with study areas. The country’s capital is Malabo, on Bioko Island. Sampling took place in two villages of mainland Equatorial Guinea, Ngonamanga and Miyobo (red stars). Ngonamanga (Litoral province, N 02°09′34,5″; E 009°47′54,4″) is a coastland village, isolated from the main trade routes, having an older population, whereas Miyobo (Centro Sur province, N 01°44′56,40″; E 10°10′40,05″) is a village in the interior, but closer to a developing city Niefang and nearby a main road, having a younger population [4]

According to the Unicef Report on Regular Resources, Equatorial Guinea has the highest gross national income per capita in Africa, but its economy is very vulnerable to fluctuations in world oil prices and the evolution of hydrocarbon production and is marked by strong social inequalities [5]. Equatorial Guinea has a low population density, estimated at 62 inhabitants per km^2^, mostly concentrated in the cities of Bata, Malabo, Mbini Ebebiyin and Mongomo (Fig. 1). Significant pockets of poverty still exist in rural areas and according to the Second National Economic Conference in Equatorial Guinea conducted in 2007, 76.8% of the population lived below the poverty line, with a daily income less than $2.00 USD [6].

In Equatorial Guinea, *Plasmodium* infections are among the leading causes of disease, with 1,221,495 of the population at risk for malaria infection, with over 291,700 cases estimated in 2016 (range 178,100–419,000) and the cause of 15% of the mortality among children under 5 years [7].

In 2004, in order to meet the Millennium Development Goals set by the United Nations, government implemented control measures on Bioko Island under the Bioko Island Malaria Control Project (BIMCP), aiming at substantially reducing, and ultimately eliminating malaria from the island. Following the success achieved in 2007 on Bioko island [8], measures were introduced on the mainland through the Equatorial Guinea Malaria Control Initiative (EGMCI). This programme included vector control interventions (large scale distribution of LLIN in Centro Sur and Wele-Nzas provinces, and IRS in Litoral and Kie-Ntem provinces) (Fig. 1), effective case management and extensive education and communication outreach initiatives. Despite the initial success, the EGMCI’s activities were discontinued in 2011 due to funding limitations and consequently, malaria prevalence remained high [9, 10].

Although Equatorial Guinea has been recently declared as a high income country (and, therefore, no longer eligible for funding from the Global Fund), it does not have an ongoing national malaria control programme, which poses a serious public health challenge for the mainland region and also is an obstacle to malaria elimination on Bioko Island, due to the increasing number of travellers from mainland [11].

## Methods

### Study area

Equatorial Guinea is a sub-Saharan country located in Central Africa. It consists of two regions, the mainland, called Rio Muni, bordered by Cameroon and Gabon and the island of Bioko (where the capital city of Malabo is located, Annobón, Corisco Bay, Elobey Chico and Elobey Grande). The mainland area of 26,000 km^2^ is divided in four provinces (Litoral, Centre Sur, Kié-Ntem and Wele-Nzas), with an estimated population of 1,835,270 in 2014 [12].

The major ethnic groups in Equatorial Guinea are Bubi (15%) in Bioko and Fang (80%) in Rio Muni. The minorities (5%) are Ndowes, Pygmies, Bisios, Fernandinos, Creoles and Annobonese [13]. In Rio Muni, the main language for inter-ethnic communication is Spanish (the official language), although Fang is highly used given the political and social hegemony of this group [14].

Although Equatorial Guinea has a tropical climate with two dry seasons in the continental region from December to mid-February and from July to September [15], malaria transmission is stable throughout the year [3]. Malaria is a major cause of morbidity and the fourth cause of death (5.7%), following HIV/AIDS (14.8%), lower respiratory infections (10%) and diarrheal diseases (8%) [1]. In the continental region, malaria is holoendemic, with *P. falciparum* being the dominant species and *Anopheles gambiae* sensu lato (s.l.) being considered the primary vector for malaria transmission [16, 17].

The study took place in two rural mainland villages with high malaria transmission, Ngonamanga and Miyobo. Ngonamanga is a coastal village close to Equatorial Guinea-Cameroon border, with mainly Ndowe (Kombe) population and where fishing is the primary economic activity. Miyobo is a Fang village in the interior of the country, near to Niefang city (15 km apart), with farming as the main economic activity.

### Study design

Between February and April 2013, a questionnaire was answered by all heads of households that agreed to voluntarily participate in the study, a total of 29 households in Ngonamanga and 40 in Miyobo to a total of 232 individuals (63 in Ngonamanga and 169 in Miyobo). According to the *Centro de Referencia para el Control de Endemias*, the estimated population density in October 2012 was 343 inhabitants in Miyobo and 69 in Ngonamanga. A malarial infection survey was done to all family members who agreed to participate. Two health technicians were selected from a list, together with the household to be visited in the following day. The health technicians informed the head of the household and family members and, in case of acceptance, a written consent was requested and the visit was scheduled for the next day. On the survey day, the individuals were fully identified, questionnaires answered and finger-prick blood samples collected. For each person, a rapid diagnostic test (RDT) (NADAL^®^ Malaria Test 4 species) was made, thick and thin blood films were performed, and some blood spotted onto Whatman^®^ 903 filter paper cards. According to the guidelines of the Ministry of Health, anti-malarial treatment of artesunate + amodiaquine (AS + AQ) with paracetamol was provided to patients with a positive RDT.

### Questionnaire

The questionnaire was written in Spanish but, whenever needed, interviews were conducted in the local language, i.e. Fang. Information on demographics (e.g. gender, age, number of people living in the house), malaria prevention measures (e.g. windows protection, use of mosquito net the night before, IRS history), house construction conditions (e.g. type of wall, floor, roof, presence/absence of eaves), and socioeconomic indicators (e.g. water source for domestic use, lighting, cooking, toilet facility, owning domestic animals) were collected.

### Identification of *Plasmodium* species

Giemsa-stained thick and thin blood films were examined under microscope at a 1000× magnification and parasite densities recorded as the number of parasites/μl of blood, assuming an average leukocyte count of 8000/μl (all smears were examined against 500 leucocytes prior to be declared negative).

*Plasmodium falciparum* asexual parasitaemia was classified according to the following criteria: low (< 800 parasites/μl), moderate (800–8000 parasites/μl), and high (≥ 8000 parasites/μl) similarly to what have done in Guerra et al. [4].

Parasite genomic DNA was extracted from a blood spot on filter paper (~ 50 μl) using a standard phenol–chloroform technique. Detection and identification of *Plasmodium* species were determined by nested-PCR amplification of the small subunit ribosomal RNA genes [18].

### Statistical analysis

An initial descriptive statistical analysis was performed to the variables collected in the above questionnaire, followed by Chi squared tests and Fisher’s Exact tests to determine possible associations between categorical variables. The variables used to estimate the socioeconomic status (SES) were based on the house characteristics, namely type of floor, walls, window protection, the presence of eaves, the energy source for cooking, toilet facilities, electricity, owning a bed net, and the number of people living in the house [19–22]. In order to establish the socioeconomic status of a household, a 2-step cluster analysis was the procedure used instead of a categorical Principal Component Analysis [23, 24], thus allowing for the inclusion of categorical and quantitative variables and the automatic selection of the number of clusters in the data.

Finally, two logistic regression analysis were performed at the individual and the household levels in order to investigate the relationship of a diverse set of independent variables on being diagnosed with malaria and on showing high levels of parasitaemia. Statistical analyses were performed using the IBM SPSS Statistics for Windows, version 22 [25], considering a significance level, α, of 0.05.

## Results

### Household and individual characteristics

All demographic, household and malaria variables in both villages, Miyobo and Ngonamanga are available in Additional file 1. In total, 69 households occupied by 232 individuals were surveyed (63 individuals in Ngonamanga and 169 in Miyobo). According to the *Centro de Referencia para el Control de Endemias*, the estimated population density in October 2012 was 69 in Ngonamanga and 343 inhabitants in Miyobo.

The participants’ age ranged from 6 months to 99 years-old, with 16.4, 19.4 and 64.2% of the individuals belonging to the age groups 6 months to 5 years old, from 6 to 15 years of age and > 15 years, respectively. Age distribution is different between villages (*P* < 0.001) due to an over-representation of older individuals (> 15 years) in Ngonamanga. The proportion of female participants were 52.6% with gender being independent of the participants’ residence (*P* = 0.222) (Additional file 1).

Demographic, household and malaria variables in Miyobo and Ngonamanga and their differences are presented in Additional file 1. In general, households are composed by an average number of 4.6 (SD = 3.06) and 2.3 (SD = 1.95) occupants in Miyobo and Ngonamanga, respectively, with 4.0 (SD = 2.5) and 3.1 (SD = 1.31) average number of rooms per household, respectively.

The majority of the houses have eaves (80%), walls of clay/wood (90%) and zinc roofs (99%); floor type differs by village, with cement/tile present in 33 and 79% of the houses in Miyobo and Ngonamanga, respectively (*P* = 0.002). In total, 71% of the houses have access to electricity from an oil generator that is used only in special occasions, and some families owned electrical devices as a radio (73%), television (41%), refrigerator (13%) or freezer (32%). The source of water for domestic use is mainly provided by closed reservoirs (88%). Firewood remains the most widely used source of power for cooking in Miyobo’s families, while in Ngonamanga, 69% of the families used gas or kerosene (*P* < 0.001). Only 2.5% of families had a flush toilet or a protected pit latrine in Miyobo, while in Ngonamanga 21% of families had better toilet facilities (*P* = 0.018).

Bed net ownership by household was 40% in Miyobo and 28% in Ngonamanga. Of those with access to a bed net, 36 and 19% of individuals from Miyobo and Ngonamanga (*P* = 0.016), respectively, had slept under it the night before the interview. The use of bed nets is prioritized for the 0–5 years old age group but it was higher in Ngonamanga (66%, 2 out of 3) than in Miyobo (57%, 20 out of 35). Usually one bed net was shared by two or three people (e.g., a couple and the youngest child). Less than 5% of the bed nets were treated with insecticide in both villages.

### Socioeconomic status (SES)

The results from a 2-step cluster analysis based on the house characteristics determined three levels for the SES, “Less poor”, “Poor”, and “More Poor”, with 29.0% (n = 20), 31.9% (n = 22) and 39.1% (n = 27) belonging to these categories, respectively (Table 1). All members of one household were classified according the SES classification of the house determined by this analysis.

**Table 1:** Occurrence, indicated as a percentage, of each predictor variable as determined in the 2-step cluster analysis

	Socioeconomic status					
	More poor	%	Poor	%	Less poor	%
Households	27	39.1	22	31.9	20	29.0
Average household size	2.78		5.64		2.65	
Eaves present		92.6		81.8		60
Source of water for domestic use	Low quality	88.9	Low quality	90.9	Low quality	90.0
Toilet facility	High quality	100	High quality	100	High quality	65.0
Has at least 1 electric appliance		70.4		100		100
Source of power for cooking	Low quality	92.6	Low quality	100	High quality	100
Wall type	Low quality	100	Low quality	54.5	Low quality	65.0
Floor type	Low quality	81.5	High quality	77.3	High quality	100
Roof type	High quality	100	High quality	100	High quality	95.0
Windows protection	Low quality	74.1	High quality	81.8	High quality	70.0
Bed net ownership	Low quality	85.2	High quality	68.2	Low quality	75.0

### Malaria infection rates

The prevalence of *Plasmodium* spp. infection was already reported in Guerra et al. [4]. The value estimated by nested-PCR was 71.4% in Ngonamanga and 68.0% in Miyobo, higher than the one estimated by optical microscopy (OM), 45.6 and 55.6%, respectively, or by RDT, 49.1 and 55.6%. Three *Plasmodium* species—*P. falciparum*, *Plasmodium malariae* and *Plasmodium ovale* were identified, with *P. falciparum* the predominant species occurring in 98% of the isolates from Ngonamanga and 96% from Miyobo, with 21% of mixed infections [4].

Ninety-three percent of the 158 samples tested positive for malaria by OM showed low parasitaemia (89% in Ngonamanga; 95% in Miyobo); 50 samples were found positive by PCR and negative by OM (13 in Ngonamanga and 37 in Miyobo) and 55 PCR-positive and RDT-negative (17 in Ngonamanga and 38 in Miyobo) were also assigned to the low parasitaemia group of < 800 parasites/μl. In the present study, OM revealed slightly better performance than NADAL^®^ Malaria Test 4 species as shown in Table 2 and both lose sensitivity with age (Additional file 2). When detecting *P. falciparum* in single or mixed infection as compared to PCR, OM had a sensitivity of 68.8% (95% CI 60.5–75.5), and specificity of 94.4% (95% CI 86.4–98.5) while the NADAL^®^ Malaria Test 4 species had a sensitivity of 65.6% (95% CI 57.7–72.9), and specificity of 90.3% (95% CI 81.0–96.0). Nevertheless, these differences were not statistically significant (P = 0.212).

**Table 2:** Sensitivity, specificity, predicative values of optical microscopy (OM) and NADAL^®^ Malaria Test 4 species with PCR as gold standard

	Positive PCR (n = 160)	Negative PCR (n = 72)	Sensitivity % (CI_95_)	Specificity % (CI_95_)	PPV % (CI_95_)	NPV % (CI_95_)
Microscopy						
Positive (n = 112)	108	4	68.8 (60.5–75.5)	94.4 (86.4–98.5)	96.4 (91.2–98.6)	57.6 (51.8–63.3)
Negative (n = 118)	50	68				
Missing (n = 2)	2					
NADAL test						
Positive (n = 112)	105	7	65.6 (57.7–72.9)	90.3 (81.0–96.0)	93.8 (88.0–96.8)	54.2 (48.5–59.7)
Negative (n = 120)	55	65				
		P-value = 0.212*				

*PVP* positive predictive value, *NVP* negative predictive value, *CI*_*95*_ 95% confidence interval

* DeLong’s test for two correlated ROC curves with AUC_microscopy_ = 81.6% and _AUCNADAL test_ = 80.0%

### Malaria determinants and parasitaemia levels

There was an association between the individual SES and the villages where they live (*P* < 0.001), with Ngonamanga showing lower levels of SES, i.e. the rejection of independence is due to a lower number of individuals classified as “Poor” together with an excess of them in the level “More Poor” in Ngonamanga. No significant association was found between SES and the presence of malaria infection (PCR diagnosis, *P* = 0.512), nor SES and parasitaemia level (*P* = 0.732, Fisher’s Exact test).

High levels of parasitaemia showed significant association with age (*P* = 0.007, Fisher’s Exact test) with an excess of higher parasitaemia cases in the 0–5 years old age group, and with the presence of animals around the house (P = 0.003, Fisher’s Exact test) with higher parasitaemia in individuals living with no animals.

Logistic regression analysis, controlled by age, between determinants (IRS, use of bed net and presence of animals) and PCR positive result and high parasitaemia as dependent variables (DV) at individual level is shown in Table 3. Considering a positive PCR result as DV, with the exception of age that showed a lower risk of infection for older people [OR = 0.979, 95% CI = (0.967–0.991)], none of the independent variables were found to be statistically significant, but IRS and the use of a bed net seemed to indicate a decrease in the risk of the infection. When high levels of parasitaemia was considered as DV, only the presence of animals in the vicinity of the house and age showed to be a statistically significant protective factor against high parasitaemia [OR = 0.053, 95% CI = (0.010–0.288); OR = 0.947, 95% CI = (0.907–0.989), respectively].

**Table 3:** Determinants for PCR positive result and high parasitaemia in logistic regression analysis at individual level

Response variable	PCR positive result			High parasitaemia			
	OR	95% CI	P-value	OR		95% CI	P-value
Age	0.979	0.967–0.991	*0.001*	0.947		0.907–0.989	*0.014*
IRS^a^							
No	1			1			
Yes	0.854	0.398–1.831	0.685	5.683		0.953–33.885	0.056
Bed net^b^							
No	1			1			
Yes	0.794	0.411–1.531	0.490	0.898		0.902–4.397	0.898
Animals^c^							
No	1			1			
Yes	1.150	0.534–2.477	0.721	0.053		0.010–0.288	*0.001*

Italic values indicate significance of *P* value (*P* < 0.05)

^a^Indoor residual spraying

^b^People sleeping under a bed net the night before

^c^Animals in the vicinity of the house

In Table 4, a logistic regression analysis was carried out at the household level, i.e., the value of 1 was assumed if at least one member of the household had a PCR positive or high parasitaemia levels. The household size appeared as a significant factor in both models, indicating that a larger household is associated with an increased risk of infection [OR = 4.771, 95% CI = (1.408–16.168)] and for high parasitaemia [OR = 1.475, 95% CI = (1.024–2.123)]. Once again, the presence of animals in the vicinity of the house seems to be a protective factor against high parasitaemia [OR = 0.070, 95% CI = (0.007–0.680)].

**Table 4:** Determinants for PCR positive result in logistic regression analysis at household level (at least one member of the household was PCR-positive) and high parasitaemia level

Response variable	PCR positive result			High parasitaemia		
	OR	95% CI	P-value	OR	95% CI	P-value
IRS^a^						
No	–	–	–	1		
Yes	–	–	–	5.071	0.733–35.053	0.100
At least Bed net used^b^						
No	1			1		
Yes	0.776	0.126–4.773	0.785	0.860	0.147–5.046	0.868
Animals^c^						
No	1			1		
Yes	3.522	0.787–15.756	0.100	0.070	0.007–0.680	*0.022*
Household size	4.771	1.408–16.168	*0.012*	1.475	1.024–2.123	*0.037*

Italic values indicate significance of *P* value (*P* < 0.05)

^a^Indoor residual spraying

^b^People sleeping under a bed net the night before

^c^Animals in the vicinity of the house

## Discussion

This study was carried out in two villages, Ngonamanga and Miyobo, of mainland Equatorial Guinea considered a high and stable malaria transmission setting [10]. Malaria prevalence and molecular characterization of *Plasmodium* populations (anti-malarial resistance-associated mutations) were previously reported [4] and allowed the analysis of results before and after the introduction of an artemisinin-based combination therapy (ACT), i.e., artesunate plus amodiaquine, 8 years apart [26]. Then, an overall prevalence of *Plasmodium* spp. infection of 69% was determined, 17% in children under 5 year-old. From the 69 studied households, 80% have at least one infected individual and the majority of cases were asymptomatic. The prevalence of malaria infections decreased by 18%, when compared to data reported by Mendes et al. [26] in the same areas, probably due to the malaria therapeutic change to AS + AQ in 2009. Although showing remarkable progress, malaria still is very prevalent, namely in such rural communities [17]. The present report intends to analyse and characterize the main malaria risk determinants in the two villages.

On a macro-scale, malaria is frequently referred as a disease of poverty. However, on a micro-scale the evidence is less consistent and more difficult to analyse and understand [27]. The present study reinforces the importance of both individual and household level factors in determining not only the risk of infection but also the risk of developing high parasitaemia in a region and a country still exposed to a very high level of malaria infection.

Equatorial Guinea still is one of the countries more affected by malaria [28]. Following the success of the BIMCP [11], malaria control has been extended to mainland Equatorial Guinea under the EGMCI but despite the moderate positive impact on the malaria infection rate, malaria control interventions on mainland are few or non-existent since 2011 [10]. According to data published by Unicef and the World Health Organization [29], 71% of rural Equatorial Guinea population uses an improved sanitation facility, and 31% uses an improved drinking water source. In this study, in both villages, more houses with access to some improved source of water for domestic use (88%) were found but there is no information about how safe this water was for consumption, and only 10% have basic sanitation facilities, a lower number than reported. Additionally, the majority of walls, floors, roofs, and windows were built with low quality housing materials, with both villages showing a low SES since people live in poor conditions.

Results failed to point an effect of SES either on the risk of malaria infection or on the risk of developing high parasitaemia. An high SES has been associated with a reduction in malaria infection risk [22, 27, 30], mainly due to the improvement of housing conditions, possibly with less mosquitoes inside the house, but Worrall et al. [27] reported that most studies that use material assets as a proxy for SES have failed to establish a positive relationship between asset ownership and a reduced incidence of malaria episodes at the household level. This lack of consistent socioeconomic differentials in malaria prevalence is not necessarily counterintuitive given the epidemiology of malaria transmission. On the contrary, the evidence of vulnerability of low SES groups is consistent and associated to inequities in access to prevention and treatment.

At both household and individual level, the household size was naturally associated to an increased malaria infection risk and vulnerability to develop high parasitaemia, corroborating what is reported in other studies that suggest that mosquitoes are more attracted to large households due to the high concentration of mosquito-attracting human emanations (carbon dioxide) [22, 31]. The studied areas showed reduced rates of IRS coverage (9%) and LLIN ownership (35%), and none of these preventive tools showed any significant effect on malaria risk. LLINs are infrequently used and those in use showed poor quality. During interviews, it was noticed that in some households bed nets were not hung onto a bed because people did not know how to use them; in others, bed nets were found in bad condition with visible holes.

Also to be noted from the results is that, despite their low application, most of the prevention measures were indoor whereas the probability of mosquito biting is mainly outdoor, thus those measures may not be very effective. As an example, this is shown by the fact that multivariate analysis at both individual and household levels suggested that the presence of animals around the house reduced the odds for a high parasitaemia. Animals may draw mosquitoes away from humans, reducing their exposure to malaria and protecting against high parasitaemia due to superinfection; alternatively, their presence may improve availability of blood meals, increasing vector density and longevity [32–34].

Entomological studies in the region suggest that *An. gambiae* s.l., *Anopheles funestus*, *Anopheles moucheti* and *Anopheles nili* s.l. are vectors of malaria in Rio Muni with *An. gambiae* sensu stricto (s.s.) being the major vector [35]. Although *An. gambiae* s.s. is usually defined as an endophagic and endophilic mosquito, exophagy and opportunistic feeding on animals have been observed in *An. gambiae* s.s. when human hosts are less available [36, 37]. Reddy et al. [38] have recently reported that malaria vectors on Bioko Island experienced a shift in host-seeking behaviour and approximately 40% of *An. gambiae* s.l. showed exophagic tendencies. Further entomological evaluation of local vector populations is required to clarify this effect.

The education level, a key factor for a sustainable response against malaria [39], has not been evaluated in this study. The knowledge about malaria transmission and prevention is crucial to improve living conditions and house construction in rural areas, which has an impact in endemic vector borne diseases. Recent Equatorial Guinea statistical information shows that in 2012, net enrolment in primary education was only 61%, and probably this number is lower in rural populations [40]. Romay-Barja et al. [41] studied individual knowledge about malaria in Bata district (urban and rural areas) and reported that almost 65% of the individuals living in the rural areas were unaware of how malaria is transmitted and the use of bed nets were mentioned more to prevent mosquito nuisance than for malaria prevention and this lack of information about malaria prevention tools and practices was frequent in both villages.

Though the discovery of oil creates an opportunity to enhance the country infrastructure and to improve the general well-being of citizens, the oil reserves are currently on the brink of being exhausted. As a part of the Horizon 2020, the Government adopted the National Plan for Economic and Social Development, which targets economic diversification and poverty reduction. The first phase, focused on infrastructure development, was concluded in 2012. The second phase will focus on economic diversification and ensure sustainability of the country economy, targeting strategic new sectors such as fisheries, agriculture, tourism, and finance [40, 42].

With the economy instability, countries tend to abandon malaria control programmes, a fact that occurred in Equatorial Guinea since 2011. After the success observed with BIMCP, to know the environmental conditions of the villages is of the utmost importance in order to design and implement effective measurements in accordance with the economic inequalities present in the continental area.

## Conclusion

This study has contributed to reinforce the importance of living conditions associated to a high risk of malaria infection and vulnerability to develop high parasitaemia. This study also contributes to future malaria control interventions to be implemented in mainland Equatorial Guinea or in other countries with similar environmental conditions.

## Additional files

**Additional file 1.** Demographic, household and malaria variables in Miyobo and Ngonamanga.

**Additional file 2.** Sensitivity, specificity and predictive values of optical microscopy (OM) and NADAL^®^ Malaria Test 4 species by age group with PCR as gold standard.

## Abbreviations

ACT: artemisinin-based combination therapy
AQ: amodiaquine
AS: artesunate
BIMCP: Bioko Island Malaria Control Project
CI: confidence interval
DV: dependent variable
EGMCI: Equatorial Guinea Malaria Control Initiative
IRS: indoor residual spraying
LLIN: long-lasting insecticide-treated net
OM: optical microscopy
OR: odds ratio
P: P-value
PCR: polymerase chain reaction
RDT: rapid diagnostic test
SD: standard deviation
SES: socioeconomic status
