# Supplementary material for: Malaria determining risk factors at the household level in two rural villages of mainland Equatorial Guinea
Source: Malar J. 2018 May 18;17:203. doi: 10.1186/s12936-018-2354-x (PMC5960103; doi:10.1186/s12936-018-2354-x)
Supplement: Supplementary file 1 — Additional file 1. Demographic, household and malaria variables in Miyobo and Ngonamanga. [file 12936_2018_2354_MOESM1_ESM.docx]

**Additional file 1:** Demographic, household and malaria variables in Miyobo and Ngonamanga

|  | | **Quality of living conditions** | **Miyobo** | | **Ngonamanga** | | **Total** | **P-value** |
| --- | --- | --- | --- | --- | --- | --- | --- | --- |
|  |  |  | **n** | **%** | **n** | **%** |  |  |
| **DEMOGRAPHICS** | |  |  |  |  |  |  |  |
| Number of individuals | |  | 169 |  | 63 |  | 232 |  |
| Males | |  | 76 | 45.0 | 34 | 54.0 | 110 | 0.222 |
| Age | Median (years) |  | 23 |  | 40 |  | 30 |  |
|  | Age 0-5 |  | 35 | 20.7 | 3 | 4.8 | 38 | **<0.001** |
|  | Age 6-15 |  | 39 | 23.1 | 6 | 9.5 | 45 |  |
|  | Age >15 |  | 95 | 56.2 | 54 | 85.7 | 149 |  |
| **HOUSEHOLDS** | |  |  |  |  |  |  |  |
| Number of households | |  | 40 |  | 29 |  | 69 |  |
| Average number of rooms | |  | 4 (SD=2.05) |  | 3.1  (SD=1.31) |  | 3.6 (SD=1.82) |  |
| Average household size | |  | 4.6 (SD=3.06) |  | 2.3 (SD=1.95) |  | 3.7 (SD=2.87) |  |
| **Types of structure in household** | |  |  |  |  |  |  |  |
| Eaves present | |  | 34 | 85 | 21 | 72.4 | 55 | 0.199 |
| **Source of water for domestic use** | |  |  |  |  |  |  |  |
| Open (well, river) | | Low | 3 | 7.5 | 5 | 17.2 | 8 | 0.140* |
| Closed (man made reservoirs, dug well protection, piped water, bottled) | | High | 37 | 92.5 | 24 | 82.8 | 61 |  |
| **Toilet facility** | |  |  |  |  |  |  |  |
| Open area (Pit latrine, forest) | | Low | 39 | 97.5 | 23 | 79.3 | 62 | **0.018*** |
| Close area (Flush toilet, pit latrine protected) | | High | 1 | 2.5 | 6 | 20.7 | 7 |  |
| **Electricity** | |  |  |  |  |  |  |  |
| No | | Low |  |  |  |  |  | 0.450 |
| Yes | | High | 27 | 67.5 | 22 | 75.9 | 49 |  |
| **Radio** | |  |  |  |  |  |  |  |
| No | | Low |  |  |  |  |  | 0.994 |
| Yes | | High | 29 | 72.5 | 21 | 72.4 | 50 |  |
| **TV** | |  |  |  |  |  |  |  |
| No | | Low |  |  |  |  |  | 0.380 |
| Yes | | High | 18 | 45.0 | 10 | 34.5 | 28 |  |
| **Fridge** | |  |  |  |  |  |  |  |
| No | | Low |  |  |  |  |  | 0.378 |
| Yes | | High | 4 | 10.0 | 5 | 17.2 | 9 |  |
| **Freezer** | |  |  |  |  |  |  |  |
| No | | Low |  |  |  |  |  | 0.897 |
| Yes | | High | 13 | 32.5 | 9 | 31.0 | 22 |  |
| **Source of power for cooking** | |  |  |  |  |  |  |  |
| Firewood | | Low | 38 | 95.0 | 9 | 31.0 | 47 | **<0.001*** |
| Gas/ Kerosene | | High | 2 | 5.0 | 20 | 69.0 | 22 |  |
| **Type of wall** | |  |  |  |  |  |  |  |
| Clay/wood | | Low | 38 | 95.0 | 24 | 82.8 | 62 | 0.086* |
| Cement/ Zinc blocks | | High | 2 | 5.0 | 5 | 17.2 | 7 |  |
| **Type of floor** | |  |  |  |  |  |  |  |
| Earth | | Low | 22 | 55.0 | 5 | 17.2 | 27 | **0.002** |
| Cement /Tile | | High | 18 | 45.0 | 24 | 82.8 | 42 |  |
| **Type of roof** | |  |  |  |  |  |  |  |
| Wood | | Low | 0 | 0.0 | 1 | 3.4 | 1 | 0.420* |
| Zinc blocks | | High | 40 | 100.0 | 28 | 96.6 | 68 |  |
| **Windows protection** | |  |  |  |  |  |  |  |
| No | | Low | 15 | 37.5 | 15 | 51.7 | 30 | 0.239 |
| Yes | | High | 25 | 62.5 | 14 | 48.3 | 39 |  |
| **Animals around household** | |  |  |  |  |  |  |  |
| No | |  | 15 | 37.5 | 14 | 48.3 | 29 | 0.371 |
| Yes | |  | 25 | 62.5 | 15 | 51.7 | 40 |  |
| **MALARIA CONTROL VARIABLES** | |  |  |  |  |  |  |  |
| Household with at least 1 bed net | |  | 16 | 40.0 | 8 | 27.6 | 24 | 0.285 |
| IRS^b^ done in last 12 months | |  | 3 | 7.5 | 6 | 23.1 | 9 | 0.072 |
| Household with at least 1 case of malaria | |  | 31 | 77.5 | 24 | 82.8 | 55 | 0.592 |

SD, standard deviation.

^a^ Housing quality used in logistic regression model

^b^ Indoor residual spraying

*Fisher’s Exact Test
