# Supplementary material for: Malaria determining risk factors at the household level in two rural villages of mainland Equatorial Guinea
Source: Malar J. 2018 May 18;17:203. doi: 10.1186/s12936-018-2354-x (PMC5960103; doi:10.1186/s12936-018-2354-x)
Supplement: Supplementary file 2 — Additional file 2. Sensitivity, specificity and predictive values of optical microscopy (OM) and NADAL® Malaria Test 4 species by age group with PCR as gold standard. [file 12936_2018_2354_MOESM2_ESM.docx]

**Additional file 2: Sensitivity, specificity and predictive values of optical microscopy (OM) and NADAL® Malaria Test 4 species by age group with PCR as gold standard**

| Age | **Microscopy** | **Positive PCR** | **Negative PCR** | **Sensitivity %**  **(CI_95_)** | **Specificity %**  **(CI_95_)** | **PPV %**  **(CI_95_)** | **NPV %**  **(CI_95_)** |
| --- | --- | --- | --- | --- | --- | --- | --- |
| 0-5 | Positive | 24 | 1 | 92.3  (74.9-99.1) | 90.9  (58.7-99.8) | 96.0  (78.7-99.4) | 83.3  (56.6-95.1) |
|  | Negative | 2 | 10 |  |  |  |  |
| 6-15 | Positive | 33 | 0 | 80.5  (65.1-91.2) | 100  (29.2-100) | 100 | 27.3  (16.8-41.1) |
|  | Negative | 8 | 3 |  |  |  |  |
| > 15 | Positive | 51 | 3 | 56.0  (45.3-66-4) | 94.8  (85.6-98.9) | 94.4  (84.8-98.1) | 57.9  (52.0-63.6) |
|  | Negative | 40 | 55 |  |  |  |  |
|  | Missing | 2 |  |  |  |  |  |
|  | **NADAL test** |  |  |  |  |  |  |
| 0-5 | Positive | 27 | 2 | 100  (87.2-100) | 81.8  (48.2-97.7) | 93.1  (79.4-97.9) | 100 |
|  | Negative | 0 | 9 |  |  |  |  |
| 6-15 | Positive | 38 | 1 | 90.5  (77.4-97.3) | 66.7  (9.4-99.2) | 97.4  (88.4-99.5) | 33.3  (12.8-63.1) |
|  | Negative | 4 | 2 |  |  |  |  |
| > 15 | Positive | 40 | 4 | 44.0  (33.6-54.8) | 93.1  (83.3-98.1) | 90.9  (79.1-96.4) | 51.4  (46.6-56.3) |
|  | Negative | 51 | 54 |  |  |  |  |
